# Clinical value of fecal calprotectin for evaluating disease activity in patients with Crohn’s disease

**DOI:** 10.3389/fphys.2023.1186665

**Published:** 2023-06-01

**Authors:** Junrong Li, Mingyang Xu, Wei Qian, Fangmei Ling, Yidong Chen, Shuang Li, Yiyu Cheng, Liangru Zhu

**Affiliations:** Division of Gastroenterology, Union Hospital, Tongji Medical College, Huazhong University of Science and Technology, Wuhan, China

**Keywords:** fecal calprotectin, crohn’s disease, clinical value, disease activity, cutoff value

## Abstract

**Objective:** To explore the clinical value of fecal calprotectin (FC) for evaluating disease activity in patients with Crohn’s disease (CD) and its relationship with disease location.

**Methods:** Patients with CD were enrolled retrospectively, and clinical data, including FC levels, were collected. Clinical activity was assessed using the Crohn’s disease activity index (CDAI). Endoscopic activity was assessed using a simple endoscopic score for Crohn’s disease (SES-CD). The partial SES-CD (pSES-CD) was scored for the size of ulcers in each segment as defined by the SES-CD and was calculated as the sum of segmental ulcer scores.

**Results:** This study included 273 CD patients. The FC level was significantly positively correlated with the CDAI and SES-CD, with correlation coefficients of 0.666 and 0.674, respectively. The median FC levels in patients with clinical remission and mildly active and moderately–severely active disease were 41.01, 164.20, and 444.45 μg/g. These values were 26.94, 66.77, and 327.22 μg/g during endoscopic remission and mildly and moderately–severely active stages, respectively. Compared with c-reactive protein (CRP), the erythrocyte sedimentation rate (ESR), and other biomarker parameters, FC was better at predicting disease activity for CD patients. For an FC <74.52 μg/g, the area under the curve (AUC) for predicting clinical remission was 0.86, with a sensitivity of 89.47% and a specificity of 71.70%. Moreover, endoscopic remission was predicted with a sensitivity of 68.02% and a specificity of 85.53%. The AUC was 0.83, and the cutoff value was 80.84 μg/g. In patients with ileal and (ileo) colonic CD, FC was significantly correlated with the CDAI, SES-CD, and pSES-CD. The correlation coefficients were 0.711 (CDAI), 0.473 (SES-CD), and 0.369 (pSES-CD) in patients with ileal CD and 0.687, 0.745, and 0.714 in patients with (ileo) colonic CD, respectively. For patients in remission, those in the active stage, and those with large or very large ulcers, differences in FC levels were not significant between patients with ileal and (ileo) colonic CD.

**Conclusion:** FC is a reliable predictor of disease activity in patients with CD, including those with ileal CD. FC is thus recommended for the routine follow-up of patients with CD.

## 1 Introduction

Crohn’s disease (CD) is a persistent and recurrent inflammatory disorder of the gastrointestinal tract, which affects the entire digestive tract, particularly the terminal ileum and colon (Ayling and Kok, 2018; Ricciuto and Griffiths, 2019). Clinical manifestations are heterogeneous, and some patients develop complications such as intestinal stenosis, fistula, or abscess with disease progression, even requiring surgical intervention, which severely affects their quality of life (Wehkamp et al., 2016; Torres et al., 2017). The therapeutic goal is to induce and maintain remission and prevent long-term complications. Therefore, the accurate and objective assessment of disease activity is crucial.

Among common methods used to assess disease activity in patients with CD, endoscopy is used to visualize the gastrointestinal mucosa and perform a biopsy. However, this is an unpleasant experience for patients, both financially and physically, and it can lead to endoscope-related complications. Commonly used biomarkers, such as c-reactive protein (CRP) and the erythrocyte sedimentation rate (ESR), lack sensitivity and specificity. Therefore, a better biomarker for intestinal inflammation is required in clinical practice.

Calprotectin is mainly derived from neutrophils, with a small proportion from monocytes and macrophages, and is a 36 kDa protein (Kalla et al., 2014). It was first described as an L1 protein in 1980 and is a heterodimer composed of S100A8 and S100A9. The antimicrobial effects of calprotectin are associated with its ability to chelate metal ions. And its concentration in feces is approximately six times higher than that in plasma (Baumgart and Sandborn, 2012; Rokkas et al., 2018).

Fecal calprotectin (FC) is currently used for CD diagnosis, differential diagnosis, disease recurrence evaluations, and disease activity in patients (García-Sánchez et al., 2010; Caviglia et al., 2018). However, the utility of its use to evaluate disease activity, including the best thresholds, varies among studies. Moreover, whether FC can be used to evaluate patients with ileal CD remains controversial. Therefore, the aim of this study was to investigate the clinical value of FC in assessing disease activity in patients with CD and whether its level is affected by disease location.

## 2 Materials and methods

### 2.1 Study population

We retrospectively enrolled 273 patients with CD admitted to the Division of Gastroenterology at Wuhan Union Hospital between December 2020 and July 2022. Inclusion criteria were as follows: hospitalized CD patients diagnosed via endoscopy and histopathology; clinical data and biochemical parameters, including FC, CRP, ESR, hemoglobin (Hb), platelet count (PLT), white blood cell count (WBC), neutrophil percentage (N%), neutrophil-to-lymphocyte ratio (NLR), platelet-lymphocyte ratio (PLR), platelet-to-lymphocyte percentage ratio (PLpR), serum albumin (ALB), and D-dimer (D-D), detected within 1 week before endoscopy. The exclusion criteria were as follows: patients with other intestinal ulcerative diseases, including ulcerative colitis (UC), infectious (bacterial, fungal, or viral) enteritis, and intestinal tuberculosis; patients with colorectal cancer; patients treated with non-steroidal anti-inflammatory drugs or proton pump inhibitors within 4 weeks; patients with severe cardiovascular, respiratory, or urinary dysfunction; and pregnant or lactating patients. This study was approved by the Tongji Medical College Ethics Committee (Ethics No. 2022 [0827]).

### 2.2 Clinical and endoscopic activity evaluation

Clinical activity was evaluated using the Crohn’s disease activity index (CDAI). CDAI <150 was considered remission, 150–220 was mildly active, 221–450 was moderately active, and ≥450 was severely active. Based on the simple endoscopic score for Crohn’s disease (SES-CD), endoscopic activity was determined as follows: 0–2 indicated remission, 3–6 indicated mild activity, 7–15 indicated moderate activity, and ≥16 indicated severely active disease. According to the SES-CD, ulcers were classified and scored as absent (0), aphthous (1), large (0.5–2 cm) (2), or very large (>2 cm) (3). Partial SES-CD (pSES-CD) was determined based on the sum of the ulcer scores in each segment (terminal ileum, right colon, transverse colon, left colon, and rectum), which was between 0 and 15.

### 2.3 Measurement of FC levels

The first stool sample was collected in the morning and transferred to a research laboratory. FC levels were measured using a commercially available rapid kit (WIZ BIOTECH). The kit measures the FC concentration via fluorescence immunochromatography with a detection range of 10–2,400 μg/g. Therefore, all values below 10 μg/g and above 2,400 μg/g were considered equal to 10 and 2,400 μg/g, respectively.

### 2.4 Statistical analysis

Statistical analyses were performed using SPSS version 23.0. Graphs were drawn using GraphPad Prism software (version 8.0). Data conforming to a normal distribution are described as the mean ± standard deviation. The median values were used to represent data with skewed distributions. A *t*-test was used for data conforming to a normal distribution, whereas a non-parametric test was used for data conforming to a skewed distribution. We used the Mann–Whitney *U* test for nonparametric comparisons between two groups and the Kruskal–Wallis H test for comparisons among multiple groups. Categorical variables were presented as numbers and percentages. Spearman’s rank correlation was used for the correlation analysis. A receiver operating characteristic (ROC) curve was drawn to estimate the area under the curve (AUC), and the optimal cutoff level was estimated using Youden’s index. A two-tailed *p*-value < 0.05 was considered statistically significant.

## 3 Results

### 3.1 Patient characteristics

In this study, 273 CD patients (213 males and 60 females), with a median age of 29 years and a median duration of 12 months, were enrolled. According to the CDAI, 159 patients (58.24%) were in the clinical remission stage and 114 patients (41.76%) were in the active stage. According to the SES-CD, 76 patients (27.84%) were in the endoscopic remission stage, and 197 (72.16%) were in the endoscopic active stage. The baseline characteristics are shown in Table 1.

**TABLE 1:** Baseline characteristics of 273 patients with CD.

Variable	Median (IQR)/number (%)
Gender (male)	213 (78.02%)
Age (years)	29 (22, 36.5)
Median course (months)	12 (7, 36)
BMI	19.43 (17.50, 22.06)
Smoking	
Current smoker	15 (5.49%)
Former smoker	14 (5.13%)
Never	244 (89.38%)
Drinking	
Current drinker	4 (1.47%)
Former drinker	5 (1.83%)
Never	264 (96.70%)
Surgery	
Perianal surgery	76 (27.84%)
Intestinal surgery	30 (10.99%)
Both	9 (3.30%)
Montreal classification	
Age at diagnosis (years)	
A1	25 (9.16%)
A2	198 (72.53%)
A3	50 (18.32%)
Disease location	
L1	41 (15.02%)
L2	32 (11.72%)
L3	157 (57.51%)
L1+4	7 (2.56%)
L2+4	1 (0.37%)
L3+4	35 (12.82%)
Behavior	
B1	153 (56.04%)
B2	91 (33.33%)
B3	22 (8.06%)
B2+3	7 (2.56%)
p	114 (41.76%)
Treatment	
5-aminosalicylates	97 (35.53%)
Corticosteroids	19 (6.96%)
Immunomodulators	29 (10.62%)
Biologics	159 (58.24%)
Bowel resection	39 (14.29%)
Clinical activity	
Remission	159 (58.24%)
Mildly active	58 (21.25%)
Moderately active	54 (19.78%)
Severely active	2 (0.73%)
Endoscopic activity	
Remission	76 (27.84%)
Mildly active	95 (34.80%)
Moderately active	82 (30.04%)
Severely active	20 (7.33%)

Abbreviations: CD, Crohn’s disease; IQR, inter-quartile range; BMI, body mass index.

### 3.2 Correlation between the FC level and CDAI, SES-CD, and biochemical parameters in CD patients

The CDAI and SES-CD were positively correlated with the FC level, with correlation coefficients of 0.666 and 0.674, respectively. In addition, the FC level was positively correlated with CRP, ESR, PLT, WBC, N%, NLR, PLR, PLpR, and D-D and negatively correlated with Hb and ALB. Compared to that with CRP, ESR, and other biochemical parameters, FC showed the strongest correlation with CDAI and SES-CD (Table 2).

**TABLE 2:** Correlation analysis of FC levels with the CDAI, SES-CD, and biochemical parameters in patients with CD.

r	FC	CDAI	SES-CD
FC	1.000^*^	0.666^*^	0.674^*^
CDAI	0.666^*^	1.000^*^	0.545^*^
SES-CD	0.674^*^	0.545^*^	1.000^*^
CRP	0.552^*^	0.583^*^	0.560^*^
ESR	0.490^*^	0.620^*^	0.519^*^
Hb	−0.334^*^	−0.598^*^	−0.341^*^
PLT	0.477^*^	0.502^*^	0.481^*^
WBC	0.314^*^	0.248^*^	0.400^*^
N%	0.321^*^	0.423^*^	0.390^*^
NLR	0.425^*^	0.486^*^	0.449^*^
PLR	0.413^*^	0.514^*^	0.390^*^
PLpR	0.521^*^	0.578^*^	0.542^*^
ALB	−0.473^*^	−0.607^*^	−0.412^*^
D-D	0.287^*^	0.441^*^	0.294^*^

Abbreviations: FC, fecal calprotectin; CDAI, Crohn’s disease activity index; SES-CD, simple endoscopic score for Crohn’s disease; CD, Crohn’s disease; CRP, c-reactive protein; ESR, erythrocyte sedimentation rate; Hb, hemoglobin; PLT, platelet; WBC, white blood cell; N%, neutrophil percentage; NLR, neutrophil-to-lymphocyte ratio; PLR, platelet-lymphocyte ratio; PLpR, platelet-to-lymphocyte percentage ratio; ALB, albumin; D-D, D-dimer. ^*^: *p* < 0.05.

### 3.3 FC, CRP, and ESR levels based on different disease activities in CD patients

**FIGURE 1:**
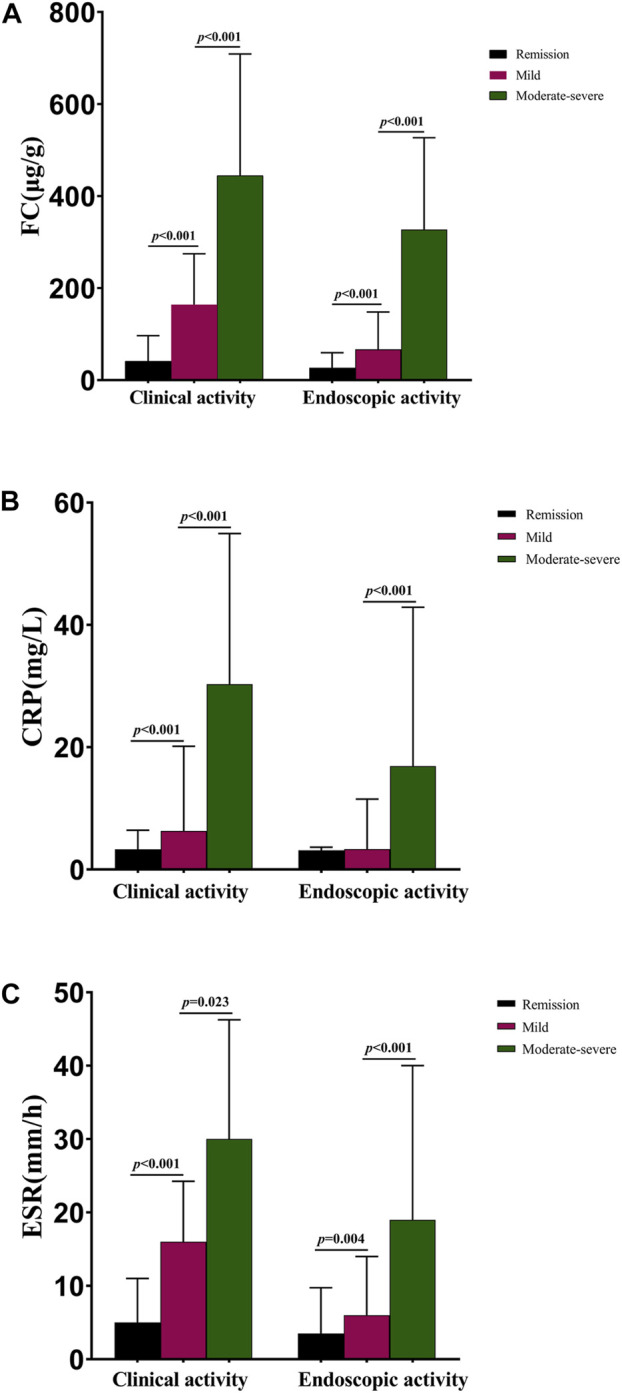
Median FC, CRP, and ESR levels in CD patients with different disease activities. **(A)** Median FC levels in CD patients with different disease activities; **(B)** Median CRP levels in patients with different disease activities; **(C)** Median ESR levels in patients with different disease activities. Abbreviations: FC, fecal calprotectin; CRP, c-reactive protein; ESR, erythrocyte sedimentation rate; CD, Crohn’s disease.

In our study, patients with severely active disease (clinically and endoscopically) were analyzed and discussed together with the moderately active group, as there were only two and 20 cases with severe clinical and endoscopic activity, respectively. As shown in Figure 1A, the median FC levels in clinical remission, mild activity, and moderate–severe activity groups were 41.01 (17.31, 96.62), 164.20 (84.49, 274.54), and 444.45 (202.47, 708.82) µg/g, with significant differences noted (*p* < 0.001). The median FC levels of CD patients in the endoscopic remission, mildly active, and moderately–severely active stages were 26.94 (10.00, 59.56), 66.77 (35.40, 147.89), and 327.22 (150.64, 526.93) µg/g, with significant differences observed (*p* < 0.001). The median CRP levels were 3.30 (2.98, 6.42), 6.30 (3.34, 20.15), and 30.30 (11.78, 54.93) mg/L in clinical remission, mildly active, and moderately–severely active groups, respectively, as shown in Figure 1B. Regarding endoscopic activity, a significant difference was observed between patients with mild activity (3.34 mg/L) and those with moderately–severely active stages (16.90 mg/L, *p* < 0.001), but not between patients with mild activity and those in remission (3.13 mg/L, *p* = 0.074). There were also significant differences in ESR among patients with different disease activities (Figure 1C). The median ESR levels were 5.00 (2.00, 11.00), 16.00 (6.00, 24.25), and 30.00 (13.25, 46.25) mm/h for clinical remission, mildly active, and moderately–severely active groups, respectively. Moreover, these values were 3.50 (2.00, 9.75), 6.00 (3.00, 14.00), and 19.00 (9.75, 40.00) mm/h for endoscopic remission, mildly active, and moderately–severely active cases, respectively. The median levels of other biochemical parameters in patients with CD with different disease activities are shown in Supplementary Tables S1A, B.

**FIGURE 2:**
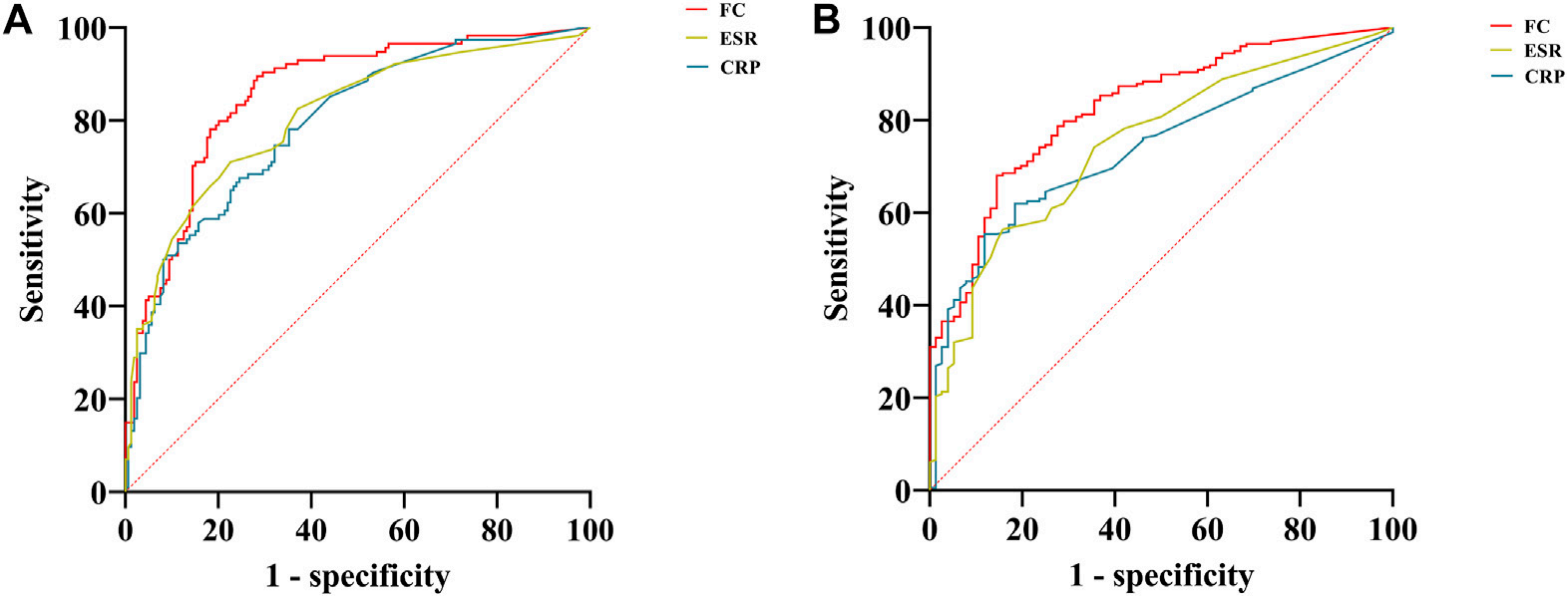
ROC curves of FC, CRP, and ESR for predicting clinical and endoscopic remission in patients with CD. **(A)** ROC curves of FC, CRP, and ESR for predicting clinical remission; **(B)** ROC curves of FC, CRP, and ESR for predicting endoscopic remission. Abbreviations: ROC, receiver operating characteristic; FC, fecal calprotectin; CRP, c-reactive protein; ESR, erythrocyte sedimentation rate; CD, Crohn’s disease.

As shown in Table 3, when the FC was <74.52 μg/g, clinical remission was predicted with a sensitivity of 89.47% and a specificity of 71.70%. When predicting endoscopic remission, the AUC was 0.83, and 80.84 μg/g was the optimal cutoff value, with a sensitivity and specificity of 68.02% and 85.53%, respectively. Compared with the CRP level and ESR, the FC level was a better predictor of clinical and endoscopic remission for CD patients. ROC curves for FC, CRP, and ESR, in terms of predicting clinical and endoscopic remission, are shown in Figures 2A, B, respectively. The ROC analysis of other biochemical parameters for the prediction of clinical and endoscopic remission is shown in Supplementary Table S2.

**TABLE 3:** ROC analysis of FC, CRP, and ESR for predicting clinical and endoscopic remission in patients with CD.

Variable		FC	CRP	ESR
Clinical remission	AUC (95% CI)	0.86 (0.81–0.90)	0.79 (0.74–0.85)	0.81 (0.75–0.86)
	Cutoff	74.52	6.46	11.5
	Sensitivity (%)	89.47	67.54	71.05
	Specificity (%)	71.70	75.47	77.37
Endoscopic remission	AUC (95% CI)	0.83 (0.77–0.88)	0.74 (0.68–0.80)	0.75 (0.69–0.81)
	Cutoff	80.84	4.21	10.5
	Sensitivity (%)	68.02%	61.93	56.35
	Specificity (%)	85.53%	81.58	84.21

Abbreviations: ROC, receiver operating characteristic; FC, fecal calprotectin; CRP, c-reactive protein; ESR, erythrocyte sedimentation rate; CD, Crohn’s disease; AUC, area under the curve.

### 3.4 Comparison of FC levels between patients with ileal and (ileo) colonic CD

Based on the Montreal classification, 41 patients with ileal CD and 189 patients with (ileo) colonic CD were enrolled in our study. As shown in Table 4, FC was positively correlated with the CDAI, SES-CD, and pSES-CD in both groups. Correlations of 0.711 and 0.687 were found between the CDAI and FC in patients with ileal and (ileo) colonic CD, respectively. In patients with (ileo) colonic CD, the correlation coefficients between FC with SES-CD or pSES-CD were 0.745 and 0.714, respectively. For patients with ileal CD, they were 0.473 and 0.369, which were weaker than those for (ileo) colonic CD patients. Table 5 shows the median FC levels based on different Montreal disease locations, which did not differ significantly for patients in remission between ileal and (ileo) colonic CD. Similarly, the median FC levels in active patients did not differ significantly. In addition, in patients with ileal and (ileo) colonic CD, the FC level in the clinically or endoscopically active stage was significantly higher than that in patients in remission. The median levels of other biochemical parameters based on different Montreal disease locations in patients with ileal and (ileo) colonic CD are shown in Supplementary Tables 3A, B.

**TABLE 4:** Correlation analysis of FC levels with CDAI, SES-CD, and pSES-CD based on different Montreal disease locations in patients with CD.

r	Ileal	(Ileo) colonic
FC & CDAI	0.711^*^	0.687^*^
FC & SES-CD	0.473^*^	0.745^*^
FC & pSES-CD	0.369^*^	0.714^*^

Abbreviations: FC, fecal calprotectin; CDAI, Crohn’s disease activity index; SES-CD, simple endoscopic score for Crohn’s disease; pSES-CD, partial SES-CD; CD, Crohn’s disease. ^*^: *p* < 0.05.

**TABLE 5:** Median level of FC based on different Montreal disease locations in patients with CD.

Activity		Ileal	(Ileo) colonic	*p*
CDAI	Remission	30.45 (15.00, 82.31)	43.15 (20.16, 101.53)	0.112
	Active	367.47 (204.99, 729.51)	236.23 (147.51, 486.18)	0.309
	*p*	<0.001	<0.001	/
SES-CD	Remission	36.81 (16.04, 140.27)	26.16 (10.00, 64.41)	0.544
	Active	203.15 (35.57, 499.26)	171.23 (63.09, 412.78)	0.966
	*p*	0.038	<0.001	/

Abbreviations: FC, fecal calprotectin; CD, Crohn’s disease; CDAI, Crohn’s disease activity index; SES-CD, simple endoscopic score for Crohn’s disease.

**FIGURE 3:**
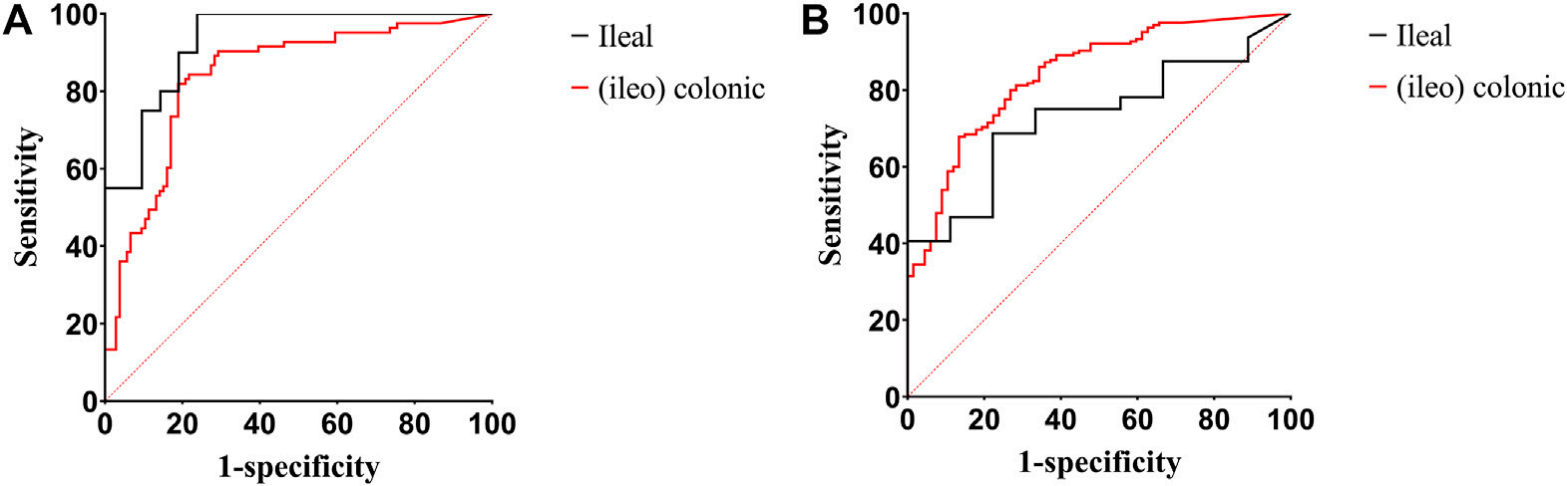
ROC curves of FC for predicting clinical and endoscopic remission in patients with ileal and (ileo) colonic CD. **(A)** ROC curves of FC for predicting clinical remission in patients with ileal and (ileo) colonic CD; **(B)** ROC curves of FC for predicting endoscopic remission in patients with ileal and (ileo) colonic CD. Abbreviations: ROC, receiver operating characteristic; FC, fecal calprotectin; CD, Crohn’s disease.

**FIGURE 4:**
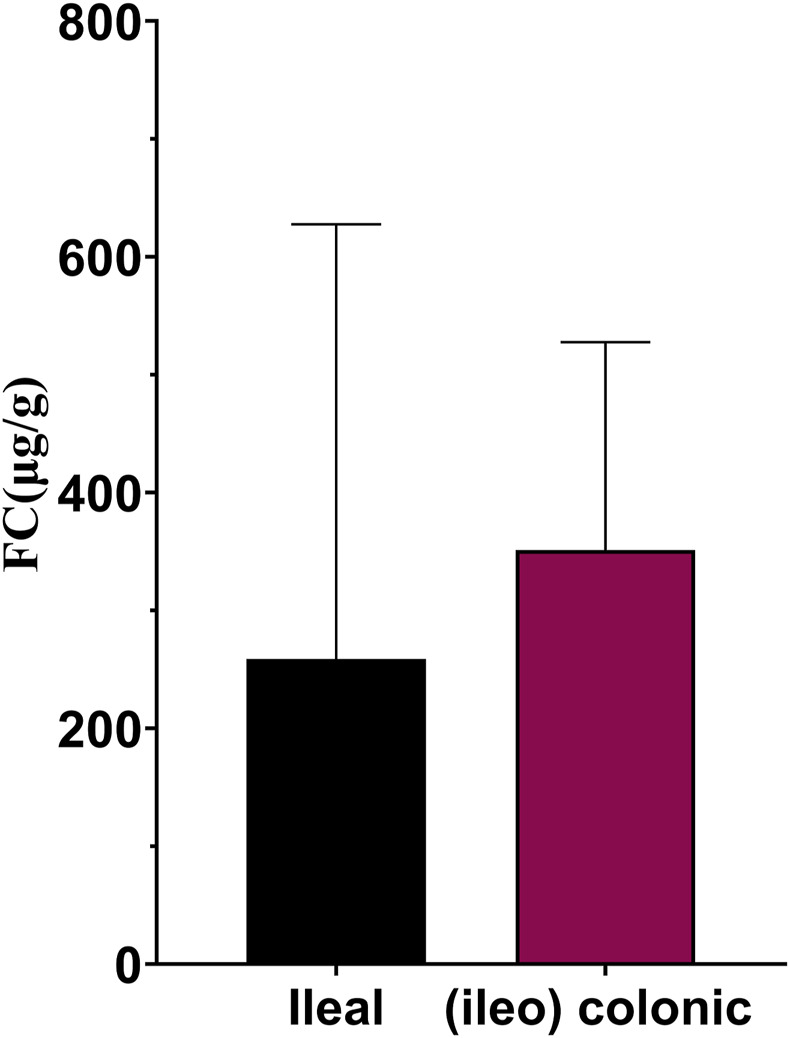
FC levels in ileal and (ileo) colonic CD in patients with large or very large ulcers. Abbreviations: FC, fecal calprotectin; CD, Crohn’s disease.

Figures 3A, B show the ROC curves of FC for the prediction of clinical and endoscopic remission in patients with ileal and (ileo) colonic CD. When predicting clinical remission in patients with ileal CD, the optimal cutoff value of FC was 59.55 μg/g. Moreover, the AUC was 0.93 (95% CI: 0.86–1.00), with a sensitivity and specificity of 100% and 76.19%, respectively. For patients with (ileo) colonic CD, the optimal cutoff value of FC was 138.90 μg/g. The AUC was 0.84 (95% CI: 0.78–0.90), with a sensitivity and specificity of 81.93% and 81.13%, respectively (Figure 3A). When predicting endoscopic remission in patients with ileal CD, the optimal FC cutoff value was 72.04 μg/g. Here, the AUC was 0.73 (95% CI: 0.57–0.89), with a sensitivity and specificity of 68.75% and 77.78%, respectively. For (ileo) colonic CD cases, the optimal cutoff value was 80.84 μg/g. Further, the AUC was 0.84 (95% CI: 0.79–0.99), with a sensitivity and specificity of 67.88% and 86.57%, respectively (Figure 3B). Among the 41 patients with ileal CD, 19 (46.34%) had large or very large ulcers with a median FC level of 258.96 (84.11, 627.51) µg/g. Among the 189 patients with (ileo) colonic CD, 78 (61.26%) had large or very large ulcers with a median FC level of 351.37 (168.17, 527.66) µg/g. The differences in the FC levels between the two groups were not significant (Figure 4).

## 4 Discussion

In recent years, FC has been gradually applied to the evaluation and management of patients with CD, including clinical and endoscopic activity evaluations, the prediction of disease recurrence, and assessing treatment responses (Boschetti et al., 2015; Veyre et al., 2021). However, the capacity of using FC to assess disease activity and whether the level of FC is affected by disease location remain controversial. Some studies indicated that FC levels in CD patients are positively correlated with endoscopic activity, with correlation coefficients of 0.6–0.8 (Sipponen et al., 2008; Schaffer et al., 2014; Iwamoto et al., 2018; Somwaru et al., 2019) and the median FC level in patients in the remission stage being lower (Schoepfer et al., 2010; Zittan et al., 2016). Moreover, compared to that with CRP, a widely used inflammatory marker, the correlation between SES-CD and FC was stronger (Matsuura et al., 2018; Knyazev et al., 2019). Different studies have reported different optimal FC thresholds for evaluating endoscopic activity. A cross-sectional study of 41 patients with CD showed that the optimal cutoff value for predicting endoscopic remission was 96 μg/g, and the sensitivity and specificity were 75.0% and 84.4%, respectively (Cannatelli et al., 2021). In another study, the sensitivity and specificity for distinguishing endoscopic remission from the active stage were 70% and 92%, respectively, with a FC level of 200 μg/g (Sipponen et al., 2008). In a study from China, a FC <156.09 μg/g predicted endoscopic remission with a sensitivity of 78.24% and a specificity of 83.61% (Han et al., 2022). The FC level when evaluating clinical activity has been reported to be significantly correlated in patients with UC but not in CD patients (D'Haens et al., 2012). However, most studies have suggested that FC can be used to evaluate clinical activity in patients with CD. For example, a meta-analysis included 727 patients with CD, and the AUC of FC for distinguishing patients in clinical remission from those in the active stage was 0.88 (Lin et al., 2014). A prospective study in Korea reported a significant correlation between the CDAI and FC in 38 CD patients (*r* = 0.520) (Lee et al., 2019). Further, a low FC level was found to predict clinical remission in CD patients, and the specificity was 100% when FC was <56 μg/g (Mooiweer et al., 2015).

In our study, the FC levels of patients in remission (26.94 μg/g) or with mild (66.77 μg/g) or moderate–severe activity (327.22 μg/g), endoscopically, differed significantly. FC could be used to predict endoscopic remission with a cutoff value of 80.84 μg/g, suggesting that endoscopy could be postponed. Further, the median FC levels were 41.01, 164.20, and 444.45 μg/g in patients in clinical remission or with mild activity or moderate–severe activity, respectively, with statistically significant differences. The cutoff value for evaluating clinical remission was found to be 74.52 μg/g, with an AUC of 0.86. The cutoff value of FC in predicting endoscopic remission was lower in our study than in other studies, which could be related to the detection method, the criteria of endoscopic activity assessment, and the subjects included. The AUC of FC for predicting clinical and endoscopic remission was higher than that of the CRP level, ESR, and other biochemical parameters, which is consistent with previous studies (Kawashima et al., 2017). In some studies, several biochemical parameters were applied to assess and predict disease activity in CD patients, such as CRP, ESR, ALB, NLR, WBC, and PLT counts (Vermeire et al., 2006; Fu et al., 2021). In our study, the SES-CD and CDAI were significantly correlated with FC, CRP, ESR, Hb, PLT, WBC, N%, NLR, PLR, PLpR, ALB, and D-D, among which the correlation with FC was the strongest, and this finding was consistent with previous studies. In addition, FC levels in patients were positively correlated with CRP levels, ESR, and WBC (Gecse et al., 2015). In our study, in addition to CRP, ESR, and WBC, FC exhibited a positive correlation with PLT, N%, NLR, PLR, PLpR, and D-D and a negative correlation with Hb and ALB.

The association between the lesion location and FC remains controversial. Some studies have suggested that the FC level in patients with ileal CD does not represent the severity of intestinal inflammation, with a weak or even no correlation (Koulaouzidis et al., 2016; Zittan et al., 2018). For example, a retrospective study showed that the sensitivity of FC in diagnosing endoscopically active stages in patients with ileal CD was only 36%, indicating that it is not a satisfactory biomarker for these patients (D'Arcangelo et al., 2021). In another study, FC levels did not increase significantly in patients with L1 CD even if prominent ulcers were observed during endoscopy (Gecse et al., 2015). However, other studies have suggested a significant correlation between FC and SES-CD in patients with ileal CD and have accurately predicted endoscopic activity (Arai et al., 2017; Monteiro et al., 2018; Romero-Mascarell et al., 2022). According to Buisson et al. (2021), FC can be used to effectively detect ulcers in patients with ileal and (ileo) colonic CD (Jensen et al., 2011). In a small prospective study in Japan, compared with that in (ileo) colonic CD patients, FC and SES-CD were more strongly correlated in patients with ileal CD (*r* = 0.78 vs. 0.67) (Inokuchi et al., 2016). In our study, FC was significantly correlated with the CDAI, SES-CD, and pSES-CD in two types of patients. However, compared with that in (ileo) colonic CD patients, the correlation among SES-CD, pSES-CD, and FC in ileal CD patients was inferior. The FC levels of patients with ileal and (ileo) colonic CD did not differ significantly in the remission or active stages. Among patients with the same lesion location, those in the active stage had significantly higher FC levels than those in remission. In addition, in patients with large or very large ulcers, the differences in FC levels were not significant between patients with ileal and (ileo) colonic CD. Therefore, FC is a reliable predictor of disease activity in patients with CD, including those with ileal CD. Nonetheless, caution should be exercised when evaluating endoscopic activity in such patients with ileal CD.

Our study has some limitations. First, owing to its retrospective nature, there is a possibility of selection bias. Second, the number of patients with clinically and endoscopically severe activity was small; therefore, the FC levels of patients in this group could not be compared and analyzed separately. Finally, the detection range of the FC level was 10–2,400 μg/g, and the FC level was <10 μg/g or >2,400 μg/g in a small number of patients, which could have influenced the results.

In conclusion, FC is a reliable predictor of disease activity in patients with CD, including those with ileal CD. Further, 74.52 μg/g and 80.84 μg/g are the best cutoff values when predicting clinical and endoscopic remission, respectively. Therefore, FC is recommended for the routine follow-up of patients with CD.

## Data Availability

The raw data supporting the conclusion of this article will be made available by the authors, without undue reservation.
