# Supplementary material for: Clinical value of fecal calprotectin for evaluating disease activity in patients with Crohn’s disease
Source: Front Physiol. 2023 Jun 1;14:1186665. doi: 10.3389/fphys.2023.1186665 (PMC10267473; doi:10.3389/fphys.2023.1186665)
Supplement: Supplementary file 1 [file DataSheet1.zip › Supplementary Table 1A.docx]

Supplementary Table 1A. Median levels of other biochemical parameters in CD patients with different clinical activities

| Variable | Clinical activity | | | *p* | |
| --- | --- | --- | --- | --- | --- |
|  | Remission | Mild | Moderate-severe | Remission vs mild | Mild vs moderate-severe |
| Hb | 134.00  (124.00, 143.00) | 118.00  (104.00, 132.00) | 109.50  (100.30, 120.00) | <0.001 | 0.322 |
| PLT | 227.00  (194.00, 265.00) | 262.00  (226.30, 334.30) | 338.50  (277.30, 395.80) | <0.001 | 0.003 |
| WBC | 5.22  (4.20, 6.57) | 5.17  (3.93, 7.08) | 6.86  (5.55, 9.60) | >0.999 | <0.001 |
| N% | 57.00  (48.20, 63.50) | 63.10  (57.73, 70.69) | 69.87  (63.05, 75.08) | <0.001 | 0.047 |
| NLR | 1.79  (1.29, 2.57) | 2.67  (2.01, 3.72) | 3.57  (2.42, 4.77) | <0.001 | 0.160 |
| PLR | 137.30  (100.00, 194.70) | 203.10  (158.00, 306.40) | 230.00  (190.00, 306.30) | <0.001 | >0.999 |
| PLpR | 7.29  (5.13, 10.35) | 10.23  (8.30, 17.14) | 16.96  (12.35, 23.05) | <0.001 | 0.008 |
| ALB | 41.80  (39.70, 45.20) | 37.50  (35.40, 40.65) | 34.45  (32.45, 38.68) | <0.001 | 0.134 |
| D-D | 0.22  (0.22, 0.27) | 0.31  (0.22, 0.59) | 0.35  (0.22, 0.59) | <0.001 | >0.999 |

Abbreviations: CD, Crohn’s disease; Hb, hemoglobin; PLT, platelet; WBC, white blood cell; N%, neutrophil percentage; NLR, neutrophil-to-lymphocyte ratio; PLR, platelet-lymphocyte ratio; PLpR, platelet-to-lymphocyte percentage ratio; ALB, albumin; D-D, D-dimer.
