# Supplementary material for: Clinical value of fecal calprotectin for evaluating disease activity in patients with Crohn’s disease
Source: Front Physiol. 2023 Jun 1;14:1186665. doi: 10.3389/fphys.2023.1186665 (PMC10267473; doi:10.3389/fphys.2023.1186665)
Supplement: Supplementary file 1 [file DataSheet1.zip › Supplementary Table 1B.docx]

Supplementary Table 1B. Median levels of other biochemical parameters in CD patients with different endoscopic activities

| Variable | Endoscopic activity | | | *p* | |
| --- | --- | --- | --- | --- | --- |
|  | Remission | Mild | Moderate-severe | Remission vs mild | Mild vs moderate-severe |
| Hb | 133.50  (122.25, 145.75) | 127.00  (117.00, 139.00) | 118.00  (104.75, 132.25) | 0.062 | 0.006 |
| PLT | 217.50  (178.50, 260.00) | 236.00  (204.00, 281.00) | 298.50  (241.25, 376.25) | 0.130 | <0.001 |
| WBC | 4.79  (3.94, 5.75) | 5.39  (4.19, 6.65) | 6.45  (5.21, 8.37) | 0.152 | <0.001 |
| N% | 55.45  (49.78, 61.99) | 59.50  (52.40, 65.40) | 68.45  (60.73, 73.30) | 0.239 | <0.001 |
| NLR | 1.73  (1.28, 2.29) | 2.00  (1.41, 2.90) | 3.17  (2.09, 4.73) | 0.240 | <0.001 |
| PLR | 135.20  (104.10, 190.70) | 159.29  (105.75, 221.01) | 225.95  (158.05, 309.73) | 0.354 | <0.001 |
| PLpR | 6.83  (4.70, 9.13) | 7.99  (5.68, 10.81) | 15.25  (10.08, 20.80) | 0.068 | <0.001 |
| ALB | 42.70  (39.80, 45.80) | 40.50  (37.40, 43.10) | 38.30  (33.65, 40.45) | 0.016 | <0.001 |
| D-D | 0.22  (0.22, 0.29) | 0.22  (0.22, 0.37) | 0.31  (0.22, 0.54) | 0.287 | 0.013 |

Abbreviations: CD, Crohn’s disease; Hb, hemoglobin; PLT, platelet; WBC, white blood cell; N%, neutrophil percentage; NLR, neutrophil-to-lymphocyte ratio; PLR, platelet-lymphocyte ratio; PLpR, platelet-to-lymphocyte percentage ratio; ALB, albumin; D-D, D-dimer.
