# Supplementary material for: Clinical value of fecal calprotectin for evaluating disease activity in patients with Crohn’s disease
Source: Front Physiol. 2023 Jun 1;14:1186665. doi: 10.3389/fphys.2023.1186665 (PMC10267473; doi:10.3389/fphys.2023.1186665)
Supplement: Supplementary file 1 [file DataSheet1.zip › Supplementary Table 2.docx]

Supplementary Table 2. ROC analysis of other biochemical parameters for predicting clinical and endoscopic remission in patients with CD

| Variable | Clinical remission | | | | Endoscopic remission | | | |
| --- | --- | --- | --- | --- | --- | --- | --- | --- |
|  | AUC  (95%CI) | Cutoff | Sensitivity  (%) | Specificity  (%) | AUC  (95%CI) | Cutoff | Sensitivity  (%) | Specificity  (%) |
| Hb | 0.80 (0.74-0.85) | 121.5 | 71.05 | 80.50 | 0.67 (0.60-0.74) | 130.5 | 64.47 | 60.53 |
| PLT | 0.75 (0.69-0.81) | 273 | 63.16 | 77.36 | 0.70 (0.64-0.77) | 222.5 | 75.63 | 56.58 |
| WBC | 0.62 (0.55-0.69) | 6.875 | 38.60 | 83.65 | 0.67 (0.60-0.74) | 5.51 | 58.88 | 72.37 |
| N% | 0.74 (0.68-0.80) | 60.01 | 78.07 | 61.01 | 0.69 (0.63-0.76) | 63.25 | 49.75 | 84.21 |
| NLR | 0.75 (0.69-0.81) | 2.175 | 76.32 | 66.67 | 0.69 (0.62-0.76) | 2.26 | 58.38 | 75.00 |
| PLR | 0.77 (0.71-0.82) | 152.5 | 82.46 | 60.38 | 0.67 (0.60-0.74) | 154.1 | 64.47 | 64.47 |
| PLpR | 0.79 (0.74-0.85) | 9.16 | 78.95 | 67.92 | 0.73 (0.67-0.79) | 9.37 | 60.91 | 78.95 |
| ALB | 0.82 (0.77-0.87) | 38.65 | 67.54 | 85.53 | 0.71 (0.64-0.77) | 40.65 | 64.47 | 71.05 |
| D-D | 0.73 (0.67-0.79) | 0.28 | 65.79 | 75.47 | 0.63 (0.56-0.70) | 0.39 | 31.47 | 93.42 |

Abbreviations: ROC, receiver operating characteristic; CD, Crohn’s disease; AUC, area under the curve; Hb, hemoglobin; PLT, platelet; WBC, white blood cell; N%, neutrophil percentage; NLR, neutrophil-to-lymphocyte ratio; PLR, platelet-lymphocyte ratio; PLpR, platelet-to-lymphocyte percentage ratio; ALB, albumin; D-D, D-dimer.
