# Supplementary material for: Clinical value of fecal calprotectin for evaluating disease activity in patients with Crohn’s disease
Source: Front Physiol. 2023 Jun 1;14:1186665. doi: 10.3389/fphys.2023.1186665 (PMC10267473; doi:10.3389/fphys.2023.1186665)
Supplement: Supplementary file 1 [file DataSheet1.zip › Supplementary Table 3A.docx]

Supplementary Table 3A. Median levels of other biochemical parameters based on different Montreal disease locations in patients with ileal CD

|  | Clinical activity | | | Endoscopic activity | | |
| --- | --- | --- | --- | --- | --- | --- |
|  | Remission | Active | *p* | Remission | Active | *p* |
| CRP | 3.13  (2.92, 10.50) | 9.72  (3.34, 19.38) | 0.007 | 3.13  (2.92, 3.30) | 9.07  (3.13, 19.38) | 0.006 |
| ESR | 3.00  (2.00, 7.00) | 14.00  (7.00, 31.25) | <0.001 | 3.00  (2.00, 7.50) | 11.00  (3.00, 22.50) | 0.023 |
| Hb | 137.00  (121.00, 145.00) | 115.50  (96.50, 120.75) | <0.001 | 127.00  (116.50, 141.00) | 120.50  (107.75, 139.00) | 0.707 |
| PLT | 227.00  (173.50, 287.00) | 303.50  (223.25, 368.50) | 0.018 | 187.00  (145.00, 265.00) | 251.00  (221.50, 331.75) | 0.016 |
| WBC | 4.93 (3.78, 6.61) | 4.40 (3.85, 6.82) | 0.666 | 3.86 (3.51, 4.75) | 4.95 (4.01, 7.27) | 0.024 |
| N% | 59.30  (46.60, 69.03) | 62.70  (57.15, 68.58) | 0.207 | 53.90  (44.20, 61.65) | 63.39  (56.20, 70.10) | 0.010 |
| NLR | 0.09 (0.08, 0.12) | 0.07 (0.06, 0.10) | 0.130 | 0.08 (0.06, 0.10) | 0.09 (0.07, 0.12) | 0.304 |
| PLR | 0.07 (0.05, 0.16) | 0.15 (0.07, 0.29) | 0.103 | 0.06 (0.05, 0.08) | 0.15 (0.06, 0.29) | 0.008 |
| PLpR | 0.02 (0.01, 0.05) | 0.03 (0.01, 0.08) | 0.366 | 0.02 (0.01, 0.02) | 0.04 (0.01, 0.08) | 0.053 |
| ALB | 41.60  (40.00, 44.85) | 34.10  (32.18, 38.03) | <0.001 | 41.40  (37.70, 42.50) | 38.65  (32.93, 42.73) | 0.265 |
| D-D | 0.22 (0.22, 0.28) | 0.30 (0.22, 0.56) | 0.045 | 0.23 (0.22, 0.28) | 0.24 (0.22, 0.50) | 0.298 |

Abbreviations: CD, Crohn’s disease; CRP, c-reactive protein; ESR, erythrocyte sedimentation rate; Hb, hemoglobin; PLT, platelet; WBC, white blood cell; N%, neutrophil percentage; NLR, neutrophil-to-lymphocyte ratio; PLR, platelet-lymphocyte ratio; PLpR, platelet-to-lymphocyte percentage ratio; ALB, albumin; D-D, D-dimer.
