# Supplementary material for: Clinical value of fecal calprotectin for evaluating disease activity in patients with Crohn’s disease
Source: Front Physiol. 2023 Jun 1;14:1186665. doi: 10.3389/fphys.2023.1186665 (PMC10267473; doi:10.3389/fphys.2023.1186665)
Supplement: Supplementary file 1 [file DataSheet1.zip › Supplementary Table 3B.docx]

Supplementary Table 3B. Median levels of other biochemical parameters based on different Montreal disease locations in patients with (ileo) colonic CD

|  | Clinical activity | | | Endoscopic activity | | |
| --- | --- | --- | --- | --- | --- | --- |
|  | Remission | Active | *p* | Remission | Active | *p* |
| CRP | 3.30  (2.98, 6.41) | 19.60  (4.41, 53.60) | <0.001 | 3.30  (2.98, 4.18) | 7.90  (3.30, 29.18) | <0.001 |
| ESR | 5.50  (3.00, 11.00) | 21.00  (10.00, 40.00) | <0.001 | 4.00  (2.00, 10.00) | 13.50  (6.00, 27.00) | <0.001 |
| Hb | 133.50  (124.00, 141.25) | 112.00  (103.00, 124.00) | <0.001 | 133.00  (123.00, 147.00) | 123.50  (107.75, 136.00) | <0.001 |
| PLT | 228.50  (200.75, 266.75) | 297.00  (245.00, 386.00) | <0.001 | 221.00  (180.00, 261.00) | 268.50  (226.00, 334.25) | <0.001 |
| WBC | 5.29 (4.12, 6.58) | 6.40 (5.29, 7.98) | <0.001 | 4.95 (3.93, 5.99) | 6.04 (4.72, 7.33) | <0.001 |
| N% | 57.25  (49.03, 63.37) | 68.20  (61.30, 72.80) | <0.001 | 56.70  (49.60, 62.70) | 63.75  (56.00, 71.48) | <0.001 |
| NLR | 0.10 (0.08, 0.12) | 0.10 (0.08, 0.13) | 0.452 | 0.09 (0.08, 0.12) | 0.10 (0.08, 0.12) | 0.112 |
| PLR | 0.07 (0.05, 0.12) | 0.29 (0.07, 0.75) | <0.001 | 0.06 (0.05, 0.08) | 0.14 (0.06, 0.45) | <0.001 |
| PLpR | 0.02 (0.01, 0.03) | 0.07 (0.02, 0.15) | <0.001 | 0.02 (0.01, 0.02) | 0.03 (0.01, 0.08) | <0.001 |
| ALB | 41.85  (39.50, 45.35) | 37.30  (33.50, 39.50) | <0.001 | 42.70  (39.10, 46.20) | 39.30  (35.45, 42.60) | <0.001 |
| D-D | 0.22 (0.22, 0.27) | 0.35 (0.22, 0.60) | <0.001 | 0.22 (0.22, 0.30) | 0.25 (0.22, 0.45) | 0.003 |

Abbreviations: CD, Crohn’s disease; CRP, c-reactive protein; ESR, erythrocyte sedimentation rate; Hb, hemoglobin; PLT, platelet; WBC, white blood cell; N%, neutrophil percentage; NLR, neutrophil-to-lymphocyte ratio; PLR, platelet-lymphocyte ratio; PLpR, platelet-to-lymphocyte percentage ratio; ALB, albumin; D-D, D-dimer.
